# Nickel chelation therapy as an approach to combat multi-drug resistant enteric pathogens

**DOI:** 10.1038/s41598-019-50027-0

**Published:** 2019-09-25

**Authors:** Stéphane L. Benoit, Alan A. Schmalstig, John Glushka, Susan E. Maier, Arthur S. Edison, Robert J. Maier

**Affiliations:** 1 Department of Microbiology, The University of Georgia, Athens, Georgia 30602 USA; 2 Center for Metalloenzyme Studies, The University of Georgia, Athens, Georgia 30602 USA; 3 Complex Carbohydrate Research Center, The University of Georgia, Athens, Georgia 30602 USA; 4 Present Address: Department of Microbiology & Immunology School of Medicine, University of North Carolina at Chapel Hill, Chapel Hill, NC 27599-7290 USA

**Keywords:** Antibiotics, Microbiology

## Abstract

The nickel (Ni)-specific chelator dimethylglyoxime (DMG) has been used for many years to detect, quantitate or decrease Ni levels in various environments. Addition of DMG at millimolar levels has a bacteriostatic effect on some enteric pathogens, including multidrug resistant (MDR) strains of *Salmonella* Typhimurium and *Klebsiella pneumoniae*. DMG inhibited activity of two Ni-containing enzymes, *Salmonella* hydrogenase and *Klebsiella* urease. Oral delivery of nontoxic levels of DMG to mice previously inoculated with *S*. Typhimurium led to a 50% survival rate, while 100% of infected mice in the no-DMG control group succumbed to salmonellosis. Pathogen colonization numbers from livers and spleens of mice were 10- fold reduced by DMG treatment of the *Salmonella*-infected mice. Using Nuclear Magnetic Resonance, we were able to detect DMG in the livers of DMG-(orally) treated mice. Inoculation of *Galleria mellonella* (wax moth) larvae with DMG prior to injection of either MDR *K. pneumoniae* or MDR *S*. Typhimurium led to 40% and 60% survival, respectively, compared to 100% mortality of larvae infected with either pathogen, but without prior DMG administration. Our results suggest that DMG-mediated Ni-chelation could provide a novel approach to combat enteric pathogens, including recalcitrant multi-drug resistant strains.

## Introduction

Every year, Enterobacteriaceae illnesses, including those by *Escherichia*, *Klebsiella*, *Salmonella*, *Shigella* and *Yersinia* species, cost billions of dollars in diarrheal illness treatment and lead to millions of human deaths. For instance, in 2013, the annual cost associated with non-typhoidal *Salmonella* infections alone was estimated at 3.67 billion dollars in the United States (United States Department of Agriculture). Among Enterobacteriaceae, multi-drug resistant (MDR) species pose one of the biggest public health challenges of our time. In its most recent list of pathogens that require new antibiotics, the World Health Organization (WHO) lists carbapenem-resistant Enterobacteriaceae (CRE) in its highest (critical) priority group^1^. A recent study conducted over 3 years in a French hospital found that bloodstream infections with MDR Enterobacteriaceae accounted for more than 70% of all bloodstream infections with MDR bacterial strains^2^. Resistance to drugs can emerge rapidly, and responses to these emerging public threats are slow or even non existent; for instance, there are no treatment guidelines established for patients carrying quinolone-resistant *Salmonella* Typhi strains^3^. Therefore, new avenues to disable these and related pathogens need to be explored: metal chelation, more specifically nickel (Ni) chelation, could be one of them. Indeed, nickel is required as a cofactor for several bacterial enzymes, including acireductone dioxygenase, [NiFe]-hydrogenase, glyoxalase I, superoxide dismutase and urease^4,5^. The nickel requirement for enzymes associated only with bacterial (and not host) enzymes has already led several groups of researchers to suggest nickel sequestration as a possible therapeutic target to combat several pathogens^6^. For instance, targeting nickel trafficking pathways to inactivate both the H_2_-uptake [Ni-Fe] hydrogenase and the urease in the gastric pathogen *Helicobacter pylori* has been proposed^7,8^. Similarly, a recent study identified the nickel requirement for *Cryptococcus neoformans*’s urease as the fungus’s “Achilles’ heel”^9^. Furthermore, the host defense protein human calprotectin was recently shown to sequester nickel away from two pathogens, *Staphylococcus aureus* and *Klebsiella pneumoniae*, subsequently inhibiting their respective urease activity in bacterial culture^10^. Many Enterobacteriaceae depend on nickel as a cofactor for their hydrogenase and/or urease enzymes. Hence, *Escherichia coli* and *Salmonella* species, including *S*. *enterica* serovar Typhimurium (from here on referred to as *S*. Typhimurium), possess several Ni-containing hydrogenases (but not urease), while *Klebsiella* species, such as *K. pneumoniae*, possess the urease enzyme, as well as several hydrogenases. It has been shown that molecular hydrogen (H_2_) use (by H_2_-uptake [Ni-Fe] hydrogenases Hya, Hyb and Hyd) is essential for *S*. Typhimurium virulence^11–13^. Although it has not been formally demonstrated, H_2_ metabolism is hypothesized to be equally crucial for *K. pneumoniae*’s virulence. Based on these results and predictions, it seems Ni-chelation as an antibacterial therapy approach should be pursued: this should especially be explored in possible application to MDR strains of Ni-requiring pathogens.

Some metal chelators are already used (or still under clinical trials) as drugs to control various human diseases, including cardiovascular diseases (reviewed in^14^) and Alzheimer’s disease^15^. Oral chelation is currently used to treat the hepatocellular copper inherited disorder known as Wilson disease^16^. Furthermore, metal chelators can also be used to neutralize metal toxicity^17,18^, including nickel toxicity: for instance the chelating agent sodium diethyldithiocarbamate (DCC) has been shown to be an effective drug against nickel carbonyl poisoning^19^; likewise, disulfiram, a compound which is eventually metabolized in two DCC molecules, is FDA-approved to treat nickel carbonyl poisoning. For some diseases there are clear benefits to chelation therapy, but sometimes the therapies have met with mixed results in benefiting the patient^14,16,17^; the latter is attributed in part to the toxic side effects of the chelating chemicals^17,20^. Hence, the use of “old” chelators such as ethylene diamine tetra acetate (EDTA) and 2, 3-dimercaptopropanol (BAL) is now restricted, due to their toxicity^17^. The use of disulfiram is also controversial, since it has been associated with elevated nickel levels in rat brains^21^, as well as with hepatotoxicity in humans^22^ and elevated nickel levels in body fluids of patients with chronic alcoholism^23^.

**Figure 1:**
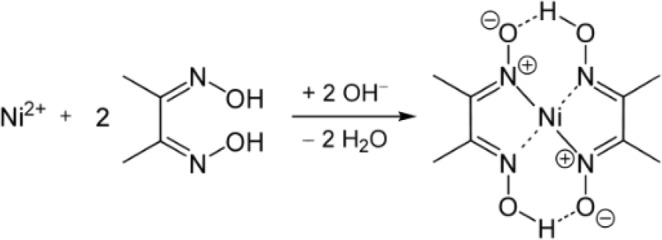
Structure of DMG and DMG-Ni. Two molecules of DMG are needed to coordinate one molecule of Ni^2+^.

In the present study, nickel-specific chelation was achieved using a commercially available chelator, dimethylglyoxime (DMG). Two molecules of DMG are needed to coordinate one Ni (II) molecule (Fig. 1). DMG stands out as it is reported to much prefer complexation with nickel over other metals. Indeed, the molecule was first described as nickel “precipitant” in 1946^24^ and was later used to identify nickel exposure of the skin^25^, a procedure commonly known as “DMG test”^26,27^. In addition, DMG is routinely used to determine nickel levels in the environment (in soil, water, industrial effluents)^28–30^ as well as to assess possible toxic levels of nickel in various items, including jewelry^31^, mobile phones^32^ or surgical items^33^. DMG has not been tested for its toxicity or as a possible therapy in animals or in humans. Finally, the Ni-specific chelator has also been used by our group and others to remove nickel from laboratory supplies, growth media, or equipment^34^, or from whole bacterial cells with the aim of studying roles of nickel in microbes. For example, studies on maturation of Ni-binding proteins (hydrogenase and/or urease) in *H. pylori*^8,35–37^ or in *Azotobacter chroococcum*^38^ have used DMG. However the impact of DMG on the growth and the subsequent *in vivo* virulence of Enterobacteriaceae had not been elucidated yet. This is the focus of the current study.

## Results and Discussion

### The nickel-specific chelator DMG inhibits growth of various enterobacteriaceae

**Figure 2:**
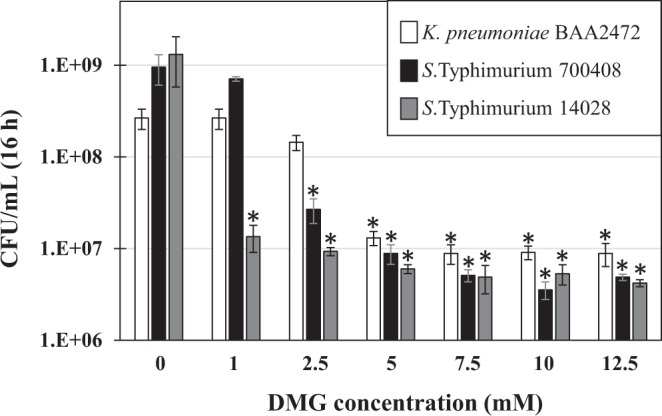
Effect of DMG on the growth of MDR *K. pneumoniae* and *S*. Typhimurium strains. *K. pneumoniae* BAA2472, *S*. Typhimurium 700408 and *S*. Typhimurium 14028 were inoculated (approximately 5 × 10^6^ CFU/mL) in appropriate media containing increasing concentrations of DMG, as indicated, and grown at 37 °C under aerobic conditions and constant shaking. Cell growth (CFU/mL) at 16 h was determined by serial dilution and plating. Results shown represent means and standard deviations from three biological replicates. The asterisk above each bar indicates the bacterial growth (CFU/mL) is significantly lower compared to the no (0) DMG control (*P* < 0.01, Student’s *t*-test).

The inhibitory effect of DMG on bacterial growth was tested on three strains: MDR *K. pneumoniae* (ATCC BAA2472), MDR *S*. Typhimurium (ATCC 700408) and *S*. Typhimurium ATCC 14028, which is a mouse colonizing strain^39^. Cells were inoculated at a starting OD_600_ of 0.005 (approximately 5 × 10^6^ CFU/mL) and grown for 16 h at 37 °C under aerobic conditions with vigorous shaking in presence of defined concentrations of DMG, as indicated, and the growth yield (CFU/mL) was determined after serial dilutions and plating (Fig. 2). The minimal DMG concentration showing significant growth inhibition (*P* < 0.01, Student’s *t*-test) for MDR *K.p*, MDR *S*.T. and *S*.T 14028 was 5 mM, 2.5 mM and 1 mM, respectively. Since approximately 5 × 10^6^ to 9 × 10^6^ cells per mL were still detected at higher DMG concentrations, our results suggest DMG has a bacteriostatic effect (rather than bactericidal) on the growth of these strains. Thus, millimolar concentrations of DMG can inhibit *in vitro* growth of various Enterobacteriaceae, including MDR strains of *K. pneumoniae* and *S*. Typhimurium.

### DMG abolishes hydrogenase activity in two *Salmonella* strains

To study the effect of Ni-chelation on hydrogenase activity in *S*. Typhimurium, cells from *S*. Typhimurium strain 14028 were grown for 6 h on blood-based media containing increasing concentrations of DMG, under H_2_-enriched microaerobic atmosphere; these conditions (blood medium and H_2_) have been previously shown to be favorable for the expression of all three *S*. Typhimurium respiratory hydrogenases^11^. No apparent growth inhibition was observed under any of the DMG concentrations, except for cells grown in presence of 10 mM DMG (reduced growth). Hydrogenase assays were carried out on whole cells using an amperometric method, as previously described^11,13^. While addition of 0.1 mM DMG to the medium had no effect on the (combined) H_2_-uptake hydrogenase activity, supplementation of the growth medium with either 0.5, 1 or 5 mM DMG significantly decreased hydrogenase activity, and addition of 10 mM DMG abolished hydrogenase activity (Table 1). The addition of 50 μM NiCl_2_ to a medium containing 1 mM DMG restored some hydrogenase activity (50% increase compared to the 1 mM DMG medium). Using a different type of hydrogenase assay (methyl viologen-based spectrophotometry, see experimental procedures), we also observed DMG-mediated inhibition of hydrogenase activity in the MDR *S*. Typhimurium strain 700408, starting with 0.5 mM DMG (data not shown). Taken together, our results indicate that DMG inhibits hydrogenase activity in *S*. Typhimurium, including in the MDR strain 700408, probably through Ni-chelation. Studies from our lab and others have shown that H_2_-uptake hydrogenase activity is required for colonization in a murine model^11–13^. Hence, the inhibitory effect of DMG on hydrogenase activity observed herein suggests the Ni-chelator could inhibit *S*. Typhimurium growth in animals.

**Table 1:** Effect of DMG chelation on hydrogenase activity in *S*. Typhimurium 14028.

DMG (mM)^*a*^	NiCl_2_ (mM)	Hydrogenase activity^*b*^
0		14.7 ± 2.8
0.1		14.0 ± 2.7
0.5		4.6 ± 1.2
1		3.9 ± 0.9
1	0.05	6.9 ± 1.7
5		0.8 ± 0.1
10		ND

^a^DMG was added to a blood-based medium, and cells were grown for 6 h under H_2_-enriched microaerobic conditions before being harvested.

^*b*^nanomoles H_2_ oxidized per min per 10^9^ cells.

Values shown are the mean ± standard deviation for 6 independent replicates. Results beginning with 0.5 mM of DMG are significantly less than without DMG (*P* < 0.01%, Student’s *t*-test).

### DMG inhibits MDR *Klebsiella pneumoniae* urease

To study the effect of Ni-chelation on urease activity in MDR *K. pneumoniae* BAA-2472, cells were grown overnight in LB broth supplemented with sublethal concentrations of DMG (no apparent growth inhibition was observed under any of the DMG concentrations), harvested and broken, and urease assays were performed on cell-free extracts (Table 2). Supplementation of the growth medium with 1 or 2 mM DMG significantly decreased urease activity in MDR *K. pneumoniae*, while addition of 5 mM completely inhibited the urease activity in the pathogenic strain. Thus, similar to hydrogenase inhibition, it appears DMG-mediated Ni chelation can be used to efficiently block urease activity in *Klebsiella*. This confirms previous results from Nakashige and coworkers, who showed that calprotectin-driven chelation of nickel led to urease inhibition in *K. pneumoniae*^10^. This is of importance, as urease is a key enzyme of nitrogen metabolism in *K. pneumoniae*. Indeed, when tested in a competition experiment with the wild-type strain, an isogenic urease mutant failed to colonize mouse intestines^40^. Thus, the inhibition of *K. pneumoniae* urease by DMG, as shown in the present study, is anticipated to have a major (inhibitory) impact on the *in vivo* colonization of the pathogen.

**Table 2:** Effect of DMG chelation on urease activity in *K. pneumoniae* BAA-2472.

DMG (mM)^*a*^	Urease activity^*b*^
0	0.17 ± 0.03
1	0.05 ± 0.01
2	0.03 ± 0.01
5	ND^*c*^

^*a*^DMG was added to LB broth, cells were grown overnight and urease assays were performed on cell-free extracts using the phenol-hypochlorite method of Weatherburn^55^.

^*b*^Urease activity is expressed in μmoles of NH_3_ produced per min per mg of total protein.

^*c*^ND, not detected (<0.001)

Values shown are the mean ± standard deviation for at least three independent biological replicates, with assays done in triplicate. Urease activities measured for all DMG-supplemented conditions are significantly lower compared to the no-DMG control (*P* < 0.01, Student’s *t*-test).

### High levels of DMG are not toxic for mice or wax moth larvae

While *in vitro* DMG-mediated inhibition of *Salmonella* and *K. pneumoniae* strains appear promising, the relatively high (millimolar) concentrations of DMG required to inhibit these pathogens’ growth could impede its *in vivo* use, due to toxicity concerns. Therefore a series of preliminary experiments were conducted to evaluate the toxicity of DMG on two animal models, *Mus musculus* (mice) and *Galleria mellonella* (greater wax moth). Mice (BALB/c) have been used (typhoid fever-mouse model) by our lab and others to study *S*. Typhimurium virulence^12,13,39^, and wax moth larvae have been proven to be a reliable model for studying virulence of many pathogens, including *K. pneumoniae*^41–43^ and *S*. Typhimurium^44–47^. Mice were subjected to various DMG treatments, including two daily doses of 0.2 mL DMG at 50 mM (~6.1 mg DMG per day) for four consecutive days. These animals displayed no obvious toxicity symptoms, as there was no apparent change in health or behavior compared to the no DMG group control over this course of chelator administration; the mice were mobile, with eyes open, they did not display any hunched posture.

A more quantitative experiment was conducted, whereby 37 day-old BALB/c mice were weighed daily for six days, upon receiving daily doses of 0.1 mL of water (control), or 0.1 ml of 100 mM DMG (3.04 mg), or 0.1 mL of 200 mM DMG (6.08 mg), on day 1 through day 5. This study confirmed that the level we used in the *Salmonella* virulence experiment (100 mM DMG, see below) is not toxic, at least in terms of affecting eating and drinking. Indeed, over the six-day period, the control mice (sterile water) gained 0.7 ± 0.2 g, while the mice receiving 100 mM DMG gained 0.9 ± 0.3 g. When 0.1 ml of 200 mM DMG (6.08 mg) was orally delivered daily to three mice, they all appeared normal in all gross parameters (mobility, posture, open eyes) and no mouse mortality or morbidity was observed; however, the weight gain data was inconclusive, since two of the mice lost weight, albeit the third mouse gained weight. In summary, orally delivered DMG does not appear to be toxic to mice under the conditions at which pathogens are inhibited. By comparison, EDTA and its derivatives have been shown to be quite toxic to animals (for a review, see^48^): for instance, the acute oral LD_50_ of Disodium EDTA in mice was found to be 400 mg/kg^49^; this corresponds to approximately 8 mg of Na_2_-EDTA for mice with an average weight of 20 g. The Ni-chelator disulfiram is even more toxic, with an oral LD_50_ of disulfiram for mice reported to be as low as 1.013 mg/kg: this corresponds to approximately 20 μg per mouse. Thus, although we did not conduct a formal toxicity study (*i.e*. LD_50_) with DMG, our results suggest oral DMG is less toxic in mice than Na_2_-EDTA, and far less toxic that disulfiram.

The toxicity of DMG was also assessed in wax moth larvae. We found that injection of 5 μl of increasing concentrations of DMG (ranging from 25 mM to 400 mM) into the rear proleg of *G. mellonella* was not detrimental to the larvae, since 70 to 100% of larvae in each group (n = 10 for each group) were still alive and active, with no change in pigmentation 72 h after injection (data not shown).

### Orally-administered DMG can be detected in mouse livers

Since DMG had never been used in animals prior to this study, the intestinal absorption and catabolism of orally delivered DMG in mice is unknown. Therefore Nuclear Magnetic Resonance (NMR) was used to detect the presence of DMG in liver samples of mice that had been given a daily oral dose (6.08 mg) of aqueous DMG for 2 to 3 days. Although NMR signals from DMG could not be detected in the aqueous supernatant from liver homogenate, diagnostic signals assigned to the methyl carbon (12.0 ppm) and protons (2.04 ppm), and to the oxime carbon (157.8 ppm) were observed in the chloroform extracts of the same supernatant (Supplemental Fig. S1, Panels A1 and A2)^50^. In contrast these signals were absent in the chloroform extracts derived from liver samples of the no DMG-control mice (Fig. S1, Panel B). Finally, DMG-specific signals were detected in chloroform extract of untreated mice upon addition of DMG to the sample (Fig. S1, Panel C). Hence, our results strongly suggest that oral DMG is intestinally absorbed and thus can be detected in the liver. This means that orally-delivered chelator has the potential to inhibit pathogens systemically.

### Oral delivery of DMG attenuates *Salmonella* virulence in mice

**Figure 3:**
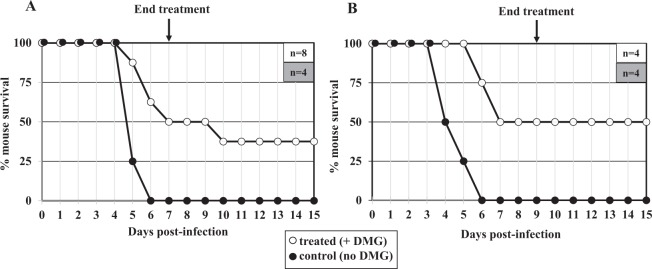
DMG-chelation attenuates *S*. Typhimurium 14028 virulence in mice. Mouse survival following infection with *S*. Typhimurium 14028 and treatment with DMG (white circles) or no DMG treatment (black circles). For DMG-treated mice, a dose of 3 mg DMG (in water) was orally given 6 h after infection with *S*.T. and then once daily, until day 7 (Panel A) or until day 9 (Panel B) post-inoculation. The last day of DMG treatment is indicated by an arrow, and the number of mice (n) used for each experiment is shown in the upper right box.

The *in vivo* efficacy of DMG against *S*. Typhimurium was assessed in mice, using the mouse-adapted *S*. Typhimurium strain 14028, as previously described^39^. In this typhoid fever-mouse model, the outcome of oral infection with *S*. Typhimurium is reproducible, typically resulting in a 100% mortality rate within a week^11–13^. This was confirmed herein: oral infection of mice with 10^6^ bacterial cells (and no DMG) led to 100% mortality within 6 days in three independent experiments (Fig. 3 and data not shown, *N* = 12 total). To determine the efficacy of DMG, a 3-mg dose of the Ni-chelator was orally delivered to mice 6 h post-infection and the same treatment (*e.g*. single daily inoculation of 3 mg DMG) was repeated every day for 3 days (data not shown), 7 days (Fig. 3A) or 9 days (Fig. 3B). The 3-day DMG treatment postponed for 2 days the time of death, however it did not change the final outcome (*e. g*. 100% mortality, data not shown). By contrast, oral delivery of DMG for 7 days (*n* = 8) and 9 days (*n* = 4) resulted in 37.5% (Fig. 3A) and 50% mouse survival (Fig. 3B), respectively. Thus, oral administration of nontoxic amounts of DMG to *S*. Typhimurium-infected mice attenuates bacterial virulence, and even leads to host survival (for up to 50% of animals). These results suggest DMG (and by extension Ni-chelation therapy) can be safely used to eradicate *S*. Typhimurium in the mouse model host.

**Figure 4:**
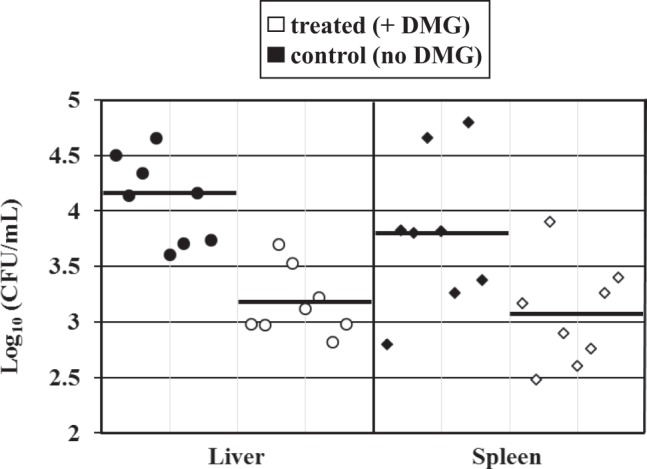
DMG treatment decreases *S*. Typhimurium organ burden in mice. Organ colonization of *S*. Typhimurium strain 14028 in the livers (circles) and spleens (diamonds) of infected mice (72 h post *S*.T. inoculation), after treatment with DMG (white symbols) or no DMG treatment (black symbols). Each symbol represents the mean (Log10) CFU/mL for one organ (liver or spleen, as indicated) and each horizontal bar represents the geometric mean of the colonization load for each group. The organ burden (mean colonization) in the DMG-treated group is significantly lower compared to the control group (no DMG), *P* < 0.01 for livers and *P* < 0.025 for spleens, respectively.

One additional experiment was performed to evaluate the effect of DMG on bacterial loads in key organs (liver and spleen) which are typically colonized in the typhoid fever-mouse model. Two groups (n = 8 each) of mice were inoculated with *S*. Typhimurium 14028; one group was orally given DMG (6 mg) three times (24 h and 30 min before infection, and 24 h post infection) while the other group did not receive any DMG. The bacterial burden in livers and spleens was determined three days after infection (Fig. 4). We found significantly lower bacterial colonization in both livers and spleens of DMG-treated animals compared to the non-DMG treated group. These results confirm the capacity of DMG to inhibit *S*. Typhimurium *in vivo*, in agreement with the lower death rate observed in DMG-treated animals.

### Injection of DMG reduces virulence of MDR enterobacteriaceae in the *Galleria mellonella* insect model

**Figure 5:**
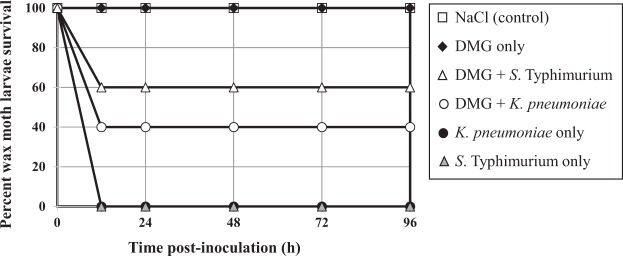
DMG-treatment attenuates virulence of MDR *K. pneumoniae* and MDR *S*. Typhimurium in the *Galleria mellonella* insect model. *G. mellonella* larvae (n = 10 for each condition) were inoculated with 5 μL of the following: 0.8% NaCl (control); 250 mM DMG; 5 × 10^5^ CFUs *K. pneumoniae* BAA2472; 250 mM DMG (left proleg) and 5 × 10^5^ CFUs *K. pneumoniae* BAA2472 (right proleg); 5 × 10^5^ CFUs *S*. Typhimurium 700408; 250 mM DMG (left proleg) and 5 × 10^5^ CFUs *S*. Typhimurium 700408 (right proleg); as indicated on the right.

Since attempts to establish a reliable *K. pneumoniae* infection model in mice failed (data not shown), an alternate animal model was chosen. As discussed above, the wax moth (*G. mellonella*) larva model has been already used to study virulence of both *K. pneumoniae* and *S*. Typhimurium, and high levels of DMG (5 μL of a 400 mM aqueous solution; approx. 0.61 mg) appear to be innocuous, as determined in this study. Injection of larvae with approximately 10^6^ CFUs of either *K. pneumoniae* and *S*. Typhimurium resulted in 100% mortality within 16 h (Fig. 5). In contrast, when DMG (5 μl of a 250 mM aqueous solution; approx. 0.38 mg) was injected 5 to 10 min prior to bacterial challenge, the treatment led to 40% and 60% survival rate for MDR *K. pneumoniae* and MDR *S*. Typhimurium, respectively. These results indicate DMG can attenuate both pathogens *in vivo* in the *G. mellonella* insect model.

In conclusion, the *in vitro* and *in vivo* results presented herein suggest that the growth of Enterobacteriaceae, including that of multi-drug resistant species of *K. pneumoniae* and *S*. Typhimurium, can be inhibited by the Ni-chelator DMG. The observed phenotype could be attributable to inhibition of important Ni-enzymes such as hydrogenase and/or urease. Other effects of DMG on cellular metabolism should not be however ruled out, and will require further work. While full growth inhibition of the pathogens required moderate to elevated levels of DMG, it is worth noting such levels of Ni-chelator appear to be innocuous when tested on two well defined animal models, *e.g*. mice and wax moth larvae. Although DMG has not been tested for its toxicity or as a possible therapy in animals or in humans, DMG-mediated chelation should be considered an alternate therapy method, especially when dealing with the ever growing threat of MDR bacterial species. Recently, the WHO released its priority pathogens list^1^. Among the twelve MDR pathogens for which new antibiotic are the most needed, ten are urease positive, six are hydrogenase positive, and four have both enzymes/activities. Furthermore, eight of those MDR species possess the Ni-containing glyoxalase I enzyme; also, five of them contain another Ni-containing enzyme, the acireductone dioxygenase. Thus, DMG-mediated targeting of either (or all) Ni-requiring enzyme(s) would prove beneficial in the fight against any of these MDR pathogenic bacteria. With this long-term goal in mind, further work is needed to understand the inhibitory effect of DMG on bacterial growth and its underlying molecular mechanisms.

## Experimental Procedures

### Bacterial strains

The MDR strain of *K. pneumoniae* subsp*. pneumoniae* used in this study was ATCC BAA-2472. It is a New Delhi metallo-beta-lactamase (NDM-1) positive strain, resistant to aminoglycosides, macrolides, fluoroquinolones, and most β-lactams, including ertapenem. *S*. Typhimurium ATCC 14028 was used for mouse colonization experiments. *S*. Typhimurium ATCC 700408 is a MDR strain, resistant to ampicillin, chloramphenicol, streptomycin, sulfonamides and tetracycline, among others.

### Chemicals

Different batches of DMG were used in this study (all from Sigma-Aldrich). For *Salmonella* hydrogenase inhibition experiments, DMG D1885 (anhydrous), was used. For growth inhibition experiments, DMG D160105 (disodium salt) was used. For all other experiments, including animal studies, DMG 40400 (disodium salt, octahydrate) was used. While DMG reference 1885 is not water soluble (methanol is required), the other two DMG preparations (D160105 and 40400) are soluble in water and aqueous buffers at concentrations up to 1.2 M, as determined in the present study.

### Growth conditions

All strains were routinely grown on LB agar plates or in LB broth. For DMG-growth inhibition study, both MDR *K. pneumoniae* (BAA-2472) and *S*. Typhimurium strain ATCC 700408 were grown in “W-U” medium, modified from Bender *et al*.^51^, containing 60 mM K_2_HPO_4_, 33 mM KH_2_PO_4_, 0.4 mM MgSO_4_, 0.4% Glucose, and 5 mM urea (instead of KNO_3_), pH 7.4. *S*. Typhimurium strain ATCC14028 was grown in W-U medium supplemented with 0.1 mM CaCl_2_ and 1 μg/mL thiamine (W-U-Ca-T). Briefly, overnight cultures of *S*. Typhimurium and *K. pneumoniae* were harvested, spun at 14,000 rpm for 5 min, washed once with W-U (MDR strains) or W-U-Ca-T (*S*.T. 14028) and resuspended in either W-U (MDR strains) or W-U-Ca-T (*S*.T. 14028). Cells were inoculated to an OD_600_ of 0.005 in W-U or W-U-Ca-T media, in presence of increasing concentrations of DMG (1, 2.5, 5, 7.5, 10 or 12.5 mM). Cells were incubated in 96 well-plates for 16 h at 37 °C with shaking at 250 rpm, then serially diluted in PBS and 5 μL of each dilution was spotted in triplicate on LB plates. Colony forming units (CFUs) were counted after 16 h at 37 °C. Each growth experiment was done 3 times.

### H_2_-uptake hydrogenase assays

Cells (from *S*. Typhimurium strains 14028 s and 700408) were grown on blood agar media for at least 6 h under a H_2_-enriched microaerobic atmosphere^11^, supplemented with only DMG (0.1, 0.5, 1, 5 or 10 mM) or 1 mM DMG and 50 μM NiCl_2_. Cells were suspended in phosphate-buffered saline (PBS) to a final concentration of 8 × 10^8^ to 1 × 10^9^ cells per ml. For *S*. Typhimurium strain 14028, hydrogenase activity was determined using an amperometric method, as previously described^52^. Briefly, cells were added to a sealed amperometric dual-electrode chamber, with constant stirring; 100 μl of H_2_-saturated phosphate-buffered saline was added to the chamber and the disappearance of H_2_ was recorded over time. Hydrogenase activity is expressed in nmoles of H_2_ oxidized per min per 10^9^ cells. Hydrogenase activity of *S*. Typhimurium strain 700408 was measured by using a modified methyl viologen (MV)-coupled spectrophotometric method^53^. Briefly, assays were conducted in a 1.8 mL sealed glass cuvette filled with a 1 mM oxidized (colorless) MV solution in PBS that had been previously sparged with pure hydrogen gas for 10 min. Small amounts of sodium dithionite were used to scavenge any residual oxygen. Once the baseline was stable, 100 μL aliquots of Triton X100-permeabilized cells^54^ were added to the cuvette and H_2_ oxidation was followed by measuring the reduction (increase in OD) of oxidized MV at 604 nm.

### Urease assays

*K. pneumoniae* cells were grown overnight at 37 °C in LB (control) or LB supplemented with 1, 2, or 5 mM DMG at 37 °C with shaking at 200 rpm. Cells were pelleted at 6,000 rpm for 30 min and washed three times with PBS (pH 7.4). Cells were standardized to an optical density (OD_600_) of 4.5, added to sterile glass beads (0.1 mm diameter, Biospec Products) at a 100% w/v ratio, and frozen at −80 °C. The bead and cell mixture was thawed at room temperature and vortexed at 3,200 rpm for 6 min with 1 min intervals on ice. The mixture was pelleted at 14,000 rpm for 2 min and the cell-free supernatant was assayed for urease activity using the phenol-hypochlorite method^55^. Protein concentration was determined using the BCA protein kit (Thermo Fisher Pierce, Rockford, IL, USA). Urease activity is expressed as μmoles of NH_3_ produced per min per mg of total protein.

### Mouse experiments

All procedures were approved by the Institutional Animal Care and Use Committee of the University of Georgia, and all procedures were performed in accordance with the relevant guidelines and regulations.

#### Chelator toxicity

A group of 8 mice were subjected to the following DMG treatment: two daily doses of 0.2 mL DMG at 50 mM (~6.1 mg DMG per day) for four consecutive days. These animals displayed no obvious toxicity symptoms. In another experiment, mice received a daily dose of 0.1 mL 40 mM DMG (~1.2 mg per day) for 4 days, 0.2 mL of 40 mM DMG (~2.4 mg per day) for four days, and then 0.2 mL of 100 mM DMG (~6.1 mg per day) for two days. Again, these mice displayed no toxicity symptoms over this course of chelator administration, or for the next three days after cessation of chelator administration (mice were then euthanized).

Three groups of 4 mice each were given 0.1 mL water (control), 0.1 mL of 100 mM aqueous DMG (~3 mg DMG) or 0.1 mL 200 mM aqueous DMG (~6.1 mg DMG) for 5 consecutive days, respectively, and their weight and mortality were monitored every day. After the fifth day, spleens and livers were harvested and analyzed for presence of DMG.

#### Detection of DMG in liver samples

A group of 8 mice was used for this experiment: 2 mice were given 0.2 mL of 100 mM DMG (~6.1 mg per day) for two days and then euthanized, and 2 mice were given the same dose for three days and then euthanized; the remaining 4 mice were used as no DMG-control. Mice were sacrificed by CO_2_ asphyxiation and cervical dislocation. Livers were quickly removed and homogenized in 2 mL sterile deionized water using a tissue homogenizer (“Tissue Tearor” model 985370, Biospec products, Bartlesville, OK, USA). Homogenized liver samples were spun at 16,800 × *g* for 6 min, and supernatants were collected before being passaged through a 0.45 μm filter unit. Filtered supernatants were subjected to NMR analysis. Since preliminary NMR experiments failed to detect DMG in individual liver samples from DMG-treated mice, these (4) samples were pooled, concentrated and also extracted with chloroform for additional NMR analysis. Liver samples from no-DMG treated mice were similarly processed and used as negative controls.

#### Infection experiment

The *in vivo* efficacy of DMG against *S*. Typhimurium was assessed by using the typhoid fever-mouse model, as previously described^39^. Female BALB/c mice (Charles River, Boston, MA) were orally inoculated individually with *S*. Typhimurium strain ATCC 14028, following previously described methods^11–13^. Briefly, *S*. Typhimurium cells grown overnight in LB were harvested, washed and suspended in sterile PBS to a final OD_600_ of 0.01 (approximately 10^7^ CFU/mL) and 0.1-ml volumes (10^6^ bacterial cells) were introduced orally into each mouse. A dose of 3 mg of DMG, corresponding to either 0.1 mL of a 100 mM or 0.2 mL of a 50 mM DMG aqueous solution, respectively, was orally given to mice belonging to one group, while the other group of mice (control) was only given sterile H_2_O. The DMG treatment was performed 6 h post-infection, then (once) every 24 h post-infection, for 3 to 9 days, as described for each experiment (three independent experiments, with n = 4 to n = 8 mice for each experiment). The mice were observed twice daily, and morbidity was recorded. In addition, a fourth independent experiment was performed to determine organ (liver and spleen) bacterial burdens. In this experiment, two groups of 8 mice each were inoculated with *S*. Typhimurium 14028. One group was treated for 3 days with one daily dose of DMG (3 mg), at 24 h and 0.5 h before infection, and 24 h post infection. Infection with *S*.T. 14028 was done as described above. Mice were euthanized 72 h after infection. Livers and spleens were removed and homogenized in sterile PBS. Dilutions of the homogenate were plated on bismuth sulfite agar (Difco-Becton Dickinson) plates, a selective medium for *Salmonella* species. Colonies (CFU) were counted after overnight incubation of the plates at 37 °C.

### *Galleria mellonella* (wax moth) larvae experiments

Wax moth larvae were obtained from local pet stores, from two different suppliers: the Bug Company (Ham Lake, MN; www.ebugco.com) and Timberline (Marion, IL; www.timberlinefresh.com). Larvae were stored in wood shavings at 4 °C in the dark and used within 2 weeks after purchase. Only larvae weighing 300 ± 50 mg were selected for the experiments. Groups of 10 larvae were used for each experiment. The site of injection (last right or left proleg) was disinfected with ethanol 70% (vol/vol) before and after each injection. A 10 μL-Hamilton syringe fitted with a 30.5-gauge needle (Beckton-Dickinson) was used to inject 5 μL of cells or DMG. After injection, larvae were kept in 9.2-cm Petri dishes at 37 °C in the dark with no food. Larvae were monitored daily for up to 4 days and mortality (as defined by change of pigmentation and absence of response following physical stimulation) was recorded.

#### Chelator toxicity

The following concentrations of DMG were tested (n = 10 for each): 5, 10, 25, 50, 75, 100, 125, 150, 200, 250, 300, 350, 400 mM. Each solution was freshly prepared in 0.8% sterile NaCl, and 5 μL of each solution was injected in the last right proleg. A control with no chelator (0.8% NaCl only) was included in the study.

#### Infection experiment

5 μL of DMG (250 mM) was injected in the last right proleg of each larva, then approximately 5 × 10^5^ bacteria (either MDR *K.p*. or MDR *S*.T.) were injected in the last left proleg 5 to 10 min after DMG inoculation. Bacterial suspensions were prepared as follows: cells were grown overnight in 5 mL of Mueller Hinton broth, harvested, washed once with and resuspended in sterile 0.8% NaCl to a final OD_600_ of 0.1 (approximately 10^8^ CFU/mL, as determined by CFU counts on serially diluted samples). Death rates were compared to those obtained after injection of NaCl only, DMG (250 mM) only, MDR *K.p*. only or MDR *S*.T. only, respectively.

### Nuclear magnetic resonance

All data were collected on a Bruker Avance Neo 800 MHz equipped with a 1.7 mM cryoprobe at 25 °C. Standard proton spectra (without and with water suppression) and two-dimensional one-bond and multiple-bond proton-carbon correlated spectra (HSQC and HMBC) were collected using Bruker pulse library sequences, zg, zgpr, hsqcetgpsp2.2, and hmbcetgpl3nd, respectively. Data were processed with Mnova software (Mestrelab, Inc.). Initial NMR samples were prepared from aqueous liver, spleen and blood preparations of individual mice, by adding 5 μL of D_2_O to a 50 μL aliquot from each sample. Since the HSQC spectrum showed no detectable DMG (data not shown), the two sets of liver samples (with DMG, no DMG) were then separately pooled, lyophilized and 100 μL D_2_O was added to the residue. Once again, no DMG was detected in the aqueous sample (data not shown). The two concentrated liver DMG samples were then extracted twice with 1 ml chloroform. The organic phase was separated and dried. 50 μL of CDCl3 was added to the residue and used for NMR analysis (see Fig. S1). NMR assignments were based on similar compounds described in Shaker^50^ and were confirmed using authentic aqueous samples of DMG and DMG + NiCl_2_ and their chloroform extracts.

## Supplementary information

Figure S1
