# Author Correction: Nickel chelation therapy as an approach to combat multi-drug resistant enteric pathogens

**DOI:** 10.1038/s41598-019-54299-4

**Published:** 2019-11-25

**Authors:** Stéphane L. Benoit, Alan A. Schmalstig, John Glushka, Susan E. Maier, Arthur S. Edison, Robert J. Maier

**Affiliations:** 1 Department of Microbiology, The University of Georgia, Athens, Georgia 30602 USA; 2 Center for Metalloenzyme Studies, The University of Georgia, Athens, Georgia 30602 USA; 3 Complex Carbohydrate Research Center, The University of Georgia, Athens, Georgia 30602 USA; 4 Present Address: Department of Microbiology & Immunology School of Medicine, University of North Carolina at Chapel Hill, Chapel Hill, NC 27599-7290 USA

Correction to: *Scientific Reports* 10.1038/s41598-019-50027-0, published online 25 September 2019

In the original version of this Article, Arthur S. Edison was incorrectly affiliated with ‘Department of Microbiology & Immunology School of Medicine, University of North Carolina at Chapel Hill, Chapel Hill, NC, 27599-7290, USA’. The correct affiliation is listed below.

Complex Carbohydrate Research Center, The University of Georgia, Athens, Georgia, 30602, USA.

This error has now been corrected in the PDF and HTML versions of the Article.

