# Supplementary material for: Nickel chelation therapy as an approach to combat multi-drug resistant enteric pathogens
Source: Sci Rep. 2019 Sep 25;9:13851. doi: 10.1038/s41598-019-50027-0 (PMC6761267; doi:10.1038/s41598-019-50027-0)
Supplement: Supplementary file 1 — Figure S1 [file 41598_2019_50027_MOESM1_ESM.pdf]

Supplementary Figure S1 for Scientific Reports SREP-19-16648

Nickel chelation therapy as an approach to combat multi-drug resistant enteric pathogens

Stéphane L. Benoit[a,b] , Alan A. Schmalstig[a], John Glushka[c], Susan E. Maier[a] , Arthur S. Edison[c] and Robert J. Maier[a,b,1]

[a]*Department of Microbiology, The University of Georgia, Athens, Georgia 30602, USA*

[b]*Center for Metalloenzyme Studies, The University of Georgia, Athens, Georgia 30602, USA*

[c]*Complex Carbohydrate Research Center, The University of Georgia, Athens, Georgia 30602, USA*

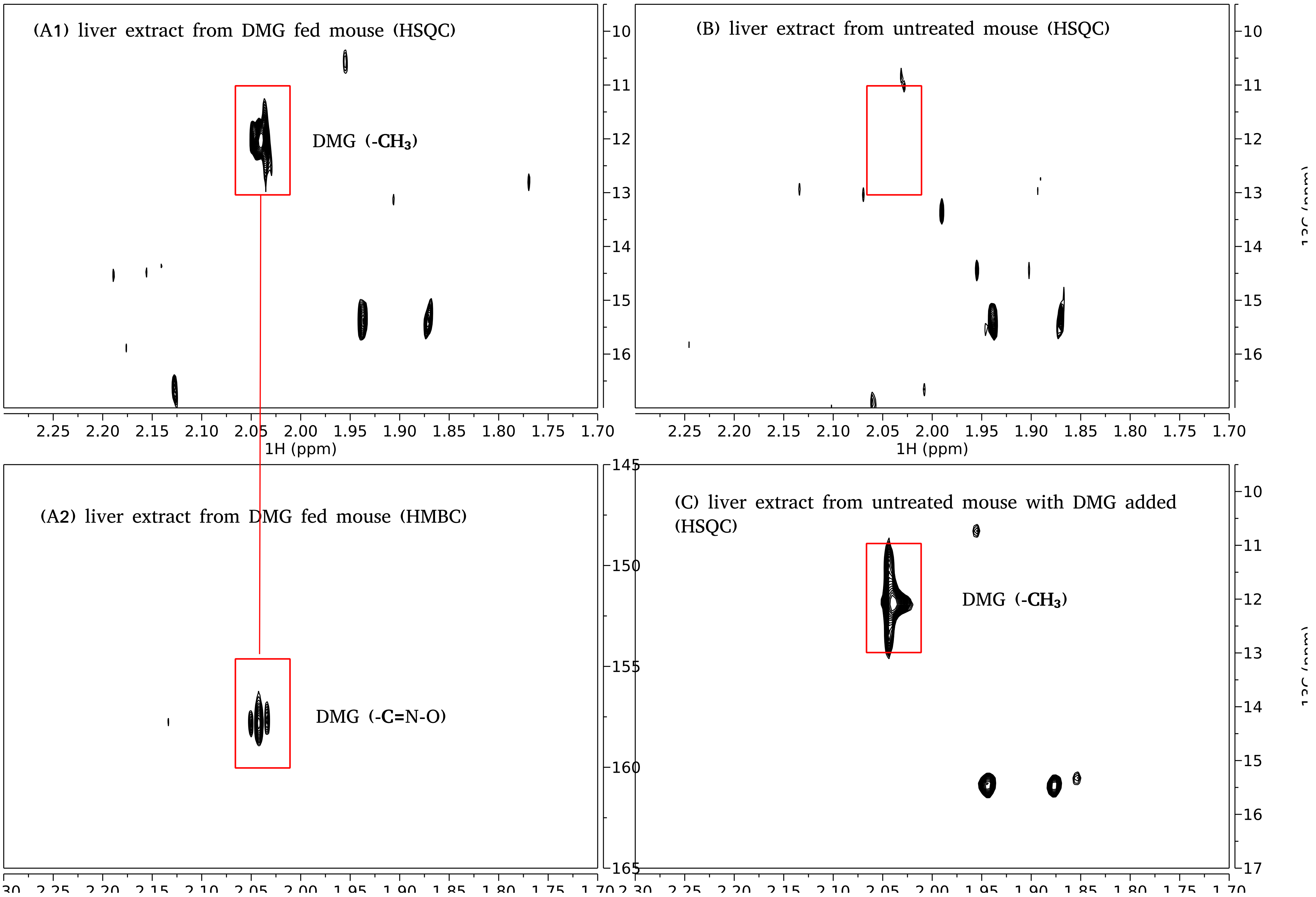
(A1) liver extract from DMG fed mouse (HSQC)
DMG (-CH3)
(B) liver extract from untreated mouse (HSQC)
(A2) liver extract from DMG fed mouse (HMBC)
DMG (-C=N-O)
(C) liver extract from untreated mouse with DMG added (HSQC)
DMG (-CH3)
1H (ppm)
1H (ppm)
2.25 2.20 2.15 2.10 2.05 2.00 1.95 1.90 1.85 1.80 1.75 1.70
10 11 12 13 14 15 16
145 150 155 160 165
10 11 12 13 14 15 16 17

Figure S1: Regions of 800 MHz 2D proton-carbon correlated spectra showing diagnostic DMG signals in liver extracts of DMG-treated mice (A) and untreated mice (B), (C).
(A1) One-bond correlated methyl protons and methyl carbon signal in HSQC spectrum.
(A2) Two-bond correlated methyl protons to oxime carbon signal.
(B) HSQC spectrum corresponding to region A1
(C) Spectrum of sample from liver extracts of untreated mice (B), with DMG added.
